# Deep Sequencing Details the Cross-over Map of Chimeric Genes in Two Porcine Reproductive and Respiratory Syndrome Virus Infectious Clones

**DOI:** 10.2174/1874357901711010049

**Published:** 2017-06-30

**Authors:** Nanhua Chen, Ranjni J. Chand, Raymond R. R. Rowland

**Affiliations:** 1 College of Veterinary Medicine, Yangzhou University, Jiangsu 225009, P.R. China.; 2 Department of Diagnostic Medicine and Pathobiology, College of Veterinary Medicine, Kansas State University, Manhattan, KS 66506, Kansas, United States.

**Keywords:** Cross-over map, Deep sequencing, Green fluorescent protein, Infectious clone, Porcine reproductive and respiratory syndrome virus (PRRSV), Viable recombinant

## Abstract

**Background:**

Recombination is an important contributor to the genetic diversity of most viruses. A reverse genetics system using green fluorescence protein (GFP)- and enhanced GFP (EGFP)-expressing infectious clones was developed to study the requirements for recombination. However, it is still unclear what types of cross-over events occurred to produce the viable offspring.

**Objective:**

We utilized 454 sequencing to infer recombination events in this system.

**Method:**

Two porcine reproductive and respiratory syndrome virus (PRRSV) infectious clones, P129-EGFP-97C and P129-GFPm-d (2-6), were co-transfected into HEK-293T cells. P129-EGFP-97C is a fully functional virus that contains a non-fluorescent EGFP. P129-GFPm-d (2-6) is a defective virus but contains a fluorescent GFPm. Successful recombination was evident by the appearance of fully functional progeny virus that expresses fluorescence. Total RNA was extracted from infected cells expressing fluorescence, and the entire fluorescent gene was amplified to prepare an amplicon library for 454 sequencing.

**Results:**

Deep sequencing showed that the nucleotide identities changed from ~37% (in the variable region from 21nt to 165nt) to 20% (T_289_C) to ~38% (456-651nt) then to 100% (672-696nt) when compared to EGFP. The results indicated that cross-over events occurred in three conserved regions (166-288nt, 290-455nt, 652-671nt), which were also supported by sequence alignments. Remarkably, the short conserved region (652-671nt) showed to be a cross-over hotspot. In addition, four cross-over patterns (two single and two double cross-over) might be used to produce viable recombinants.

**Conclusion:**

The reverse genetics system incorporating the use of high throughput sequencing creates a genetic platform to study the generation of viable recombinant viruses.

## INTRODUCTION

Recombination occurs in most RNA viruses and has a major impact on their diversification and evolution. More significantly, recombination has also been associated with the emergence of new viruses, increases in virulence and pathogenesis, and the evasion of host immune responses [1, 2]. Porcine reproductive and respiratory syndrome virus (PRRSV) is the most costly swine virus worldwide, which is an enveloped, positive-sense, single-stranded RNA virus [3]. Recombination in RNA viruses is a process to form chimeric offspring from parental genomes of mixed origin, which requires co-infection or super-infection of a cell with at least two viruses. RNA recombination is based on RNA-dependent RNA polymerase (RdRp) template switches that may occur during either genomic RNA replication or subgenomic mRNA synthesis [4]. The factors that influence template switching include RNA secondary structures and sequence similarity between donor and acceptor templates [5, 6].

Recombination is a common phenomenon between PRRSV isolates in the field [7]. The occurrence of PRRSV recombination was first suggested by the phylogenetic analysis of field isolates [8]. Among PRRSV isolates, intra-genotype recombination is frequent but no inter-genotype recombination has been reported [9-16]. PRRSV could undergo homologous recombination with the frequency from <2% up to 10% *in vitro* and ~38% (133/352) *in vivo* [12, 14]. The most widely accepted model of PRRSV recombination is copy-choice model [2, 17].

**Fig. (1):**
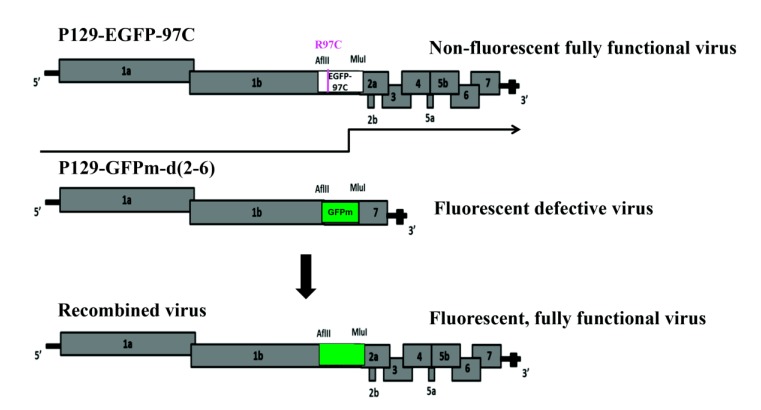
Recombination between two PRRSV infectious clones P129-EGFP-97C and P129-GFPm-d (2-6). P129-EGFP-97C is a fully functional non-fluorescent virus and P129-GFPm-d (2-6) is a fluorescent defective virus lacking ORF2-6. Successful recombination between the two parental viruses is evidenced by producing viable fluorescent progeny viruses.

Conventional methods to detect PRRSV recombination are based on PCR and sequencing followed by data analysis using recombination detection programs, such as RDP and SimPlot [18, 19]. A major limitation of these PCR based methods is that all genomic, subgenomic and defective RNAs could serve as templates for PCR amplification. Therefore, the viability of the recombinants identified by them is unknown. To address this limitation, we developed a new *in vitro* system targeting at recombination events that present in viable offspring [20]. This reverse genetics system uses green fluorescence protein (GFP)- and enhanced GFP (EGFP)-expressing PRRSV infectious clones to study recombination in the chimeric genes that are nonessential for virus replication. Successful recombination is evidenced by generating a viable fluorescent virus from the co-transfection of a non-fluorescent viable virus with a mutation in EGFP (P129-EGFP-97C) and a fluorescent defective virus (P129-GFPm-d (2-6)) (Fig. **1**). However, what types of cross-over events occurred to produce viable viruses in this system are not clarified yet. Here we took advantage of high throughput sequencing to assess the locations of all cross-over events between EGFP and GFPm genes and explore the cross-over patterns that are potentially utilized to produce viable recombined viruses.

## MATERIALS AND METHOD

### Sample Preparation

HEK-293T cells were propagated and maintained in Minimum Essential Medium Eagle (1×MEM) (Fisher Scientific) with 7% Fetal Bovine Serum (FBS) (Gibco), 80 U/ml Penicillin-Streptomycin (Gibco) and 0.3 μg/ml Fungizone Antimycotic (Gibco) at 37°C with 5% CO_2_ [21]. About 80% confluent HEK-293T cells were co-transfected with two PRRSV infectious clones: P129-EGFP-97C and P129-GFPm-d (2-6), using Fugene HD transfection reagent (Promega) according to the recommended protocol. These two infectious clones are derived from the DNA-launched P129-GFP infectious clone [22, 23] but with distinct characteristics. P129-EGFP-97C is a fully functional P129 virus with a non-fluorescent EGFP gene, the result of a C_289_T nucleotide substitution in the fluorophore active site of EGFP [24]. P129-GFPm-d (2-6) is a defective virus that lacks ORFs 2-6, but contains a fluorescent GFPm gene. GFPm is a chimeric gene that contains a middle EGFP sequence (290bp) flanked on each side by sequence derived from GFP (EGFP and GFP share only 83% nucleotide identity). Fully functional viruses that express fluorescence can be generated by recombination between these two infectious clones in fluorescent genes. Therefore, after 48 hours of co-transfection, the supernatant was used to infect 100% confluent Marc-145 cells and green fluorescent plaques could be observed at 72 hours post infection (hpi). Due to the low frequency of recombination (~0.3%), enrichment was performed using fluorescence-activated cell sorting (FACS) for cells that express green fluorescence with the MoFlo XDP Cell Sorter (Beckman Coulter). After two rounds of enrichment, roughly 80% of cells carrying the fluorescent viruses were obtained [20].

### Amplicon Library Preparation

Total RNA was extracted from 100 μl of the sorted cell sample using TRIzol® Reagent (Invitrogen) according to the manufacturer’s protocol and eluted in 50 μl RNase-free water. cDNA was generated by reverse transcription using random hexamer primers from the Transcriptor High Fidelity cDNA Synthesis Kit (Roche). Two sets of primer pairs were utilized in two rounds of PCR for the preparation of an amplicon library (Table **1**). Three overlapped regions, which were 369bp, 270bp, and 336bp in length, were amplified in the first round of PCR. A same pair of multiplex identifier (MID) primers was used for the three amplicons in the second round of PCR. The amplicon library was created by these three amplicons as we previously described [25, 26].

### Pyrosequencing

The amplicon library was sent to emPCR amplification and 454 sequencing as described previously [25, 26]. Briefly, Lib-L emPCR Kit (Roche) was used for emPCR according to the emPCR Amplification Method Manual. GS FLX Titanium Sequencing Kit XLR70 (Roche) was used for 454 sequencing following the protocol. Reads for each sample were sorted according to the MID. Sequence reads were mapped against the GFPm gene with 454 Life Sciences GS Reference Mapper (Version 2.6). Coverage was calculated and variants were called. Variants were filtered based on the coverage, variant frequency, and homopolymer. Only high confidence single nucleotide variants that have the following features were selected: (1) at least 3 non-duplicate reads have the nucleotide substitution; (2) the substitution frequency is greater than 5%; (3) the substitution is not located at homopolymer sites.

## RESULTS

### Identification of Cross-over Events

The number of reads for three amplicons were around 6300 ~ 15500. All sequences were compared to the GFPm sequence. Mutations identified by high throughput sequencing are shown in (Table **2**). The percentages of mutations identical to EGFP in the first variable region (from 21nt to 165nt) were around 31% to 41%, with 37% in average. Based on 15531 reads, only 20% of the sequences had thymine at position 289, while the other 80% were cytosine, which is identical to GFPm. The second variable region (from 456nt to 651nt) is about 29% to 45% (with the average of 38%) identical to EGFP. The third variable region (from 672nt to 696nt) has 100% identity to EGFP. The changes in the percentages of identities between variable regions after each conserved region indicated that cross-over events occurred in three conserved regions: 166-288nt, 290-455nt, and 652-671nt.

As shown in Fig. (**2**), the percentage of nucleotide identity decreased from ~37% to 20% in the 123bp-conserved region (166-288nt) when compared to EGFP, which suggested that there was a cross-over occurrence. In addition, the percentage increased from 20% to ~38% in the 166bp-conserved region (290-455nt), suggesting that another cross-over existed in this region. Remarkably, the percentage dramatically changed from 38% to 100%, which meant ~62% of the recombinant viruses proceeded cross-over in the only 20bp-conserved region (652-671nt), suggesting the 20bp-conserved region is a hotspot of cross-over.

### Potential Cross-over Patterns

Four types of cross-over events could occur to produce the recombined, viable, and fluorescent virus. As shown in Fig. (**3**), there are two types of single recombination events Figs. (**3A** and **B**), which have cross-over occurring in the 20bp-conserved region (from GFPm to EGFP) and the 166bp-conserved region (from GFPm to EGFP), respectively. Furthermore, there are other two types of double recombination events (Figs. **3C** and **D**). One has the double cross-over occurring in the 123bp-conserved region (from EGFP to GFPm) then in the 20bp-conserved region (from GFPm to EGFP), and another one occurs in the 123bp-conserved region (from EGFP to GFPm) then in the 166bp-conserved region (from GFPm to EGFP).

Although the rate of each recombination pattern could not be identified in this study, sequence alignments provided direct evidence that cross-over events occurred in the 123bp- and 20bp-conserved regions, respectively (Fig. **4**). The first representative recombinant is identical to GFPm gene before the 123bp-conserved region but becomes identical to EGFP gene from position 289 (Fig. **4A**). And the second representative recombinant is identical to GFPm gene before the 20bp-conserved region but becomes identical to EGFP gene after the conserved region (Fig. **4B**). Cross-over events occurring in the 166bp-conserved regions could not be analyzed due to the limit of read length (400bp) of 454-pyrosequencing used in our study.

## DISCUSSION

By combined utilization of the *in vitro* reverse genetics system and high throughput sequencing, we inferred recombination events between inserted fluorescent genes in PRRSV infectious clones. Based on thousands of sequences from viable progeny viruses, we found that the nucleotide identities changed between the variable and conserved regions of EGFP/GFPm genes, indicating that the cross-over events occurred in the conserved regions. The advantages of this new method in measuring recombination include: 1) This reporter system targets at detecting viable recombinant viruses. 2) The system tests recombination events in nonessential gene without affecting the virus replication. 3) The co-utilization of *in vitro* reverse genetics system and deep sequencing reveals all types of cross-over events which occurred in a target gene to produce viable recombinants. Similar reporter systems are generally used to measure the virus recombination [27, 28]. The disadvantage of using 454 pyrosequencing in our system is the introduction of errors (error rates ranged from 0.04-0.66%) [29], which resulted in the variations of the identities in a same conserved region (Table **2**); however, it did not interrupt the evaluation of the obviously changes between conserved regions. In addition, this system was developed to analyze the requirements for generating viable recombinants but not for detecting all recombination events between these two infectious clones, therefore, it could not detect the recombination events in the PRRSV genomes.

The first and second conserved regions (166-288nt and 290-455nt) are only separated by a C_289_T mutation. And the variation frequencies in two flanked variable regions (21-165nt and 456-651nt) are nearly identical (~37% and ~38%), which arouse suspicion that the lower variation frequency of C_289_T mutation (20%) might have resulted from mutation or inaccurate sequencing rather than from recombination. However, our results provide strong support that the changes in sequence identity are due to cross-over events. First, the 20% mutation rate of C_289_T results from 15531 reads (Table **2**). Second, the sequencing result of the sample (80% nucleotides at position 289 are cytosine, which is identical to the fluorescent gene GFPm) is consistent with the cell sorting result (about 80% sorted cells express fluorescence). Third, the C_289_T mutation is highly stable during serial passage in Marc-145 cells [20].

**Fig. (2):**
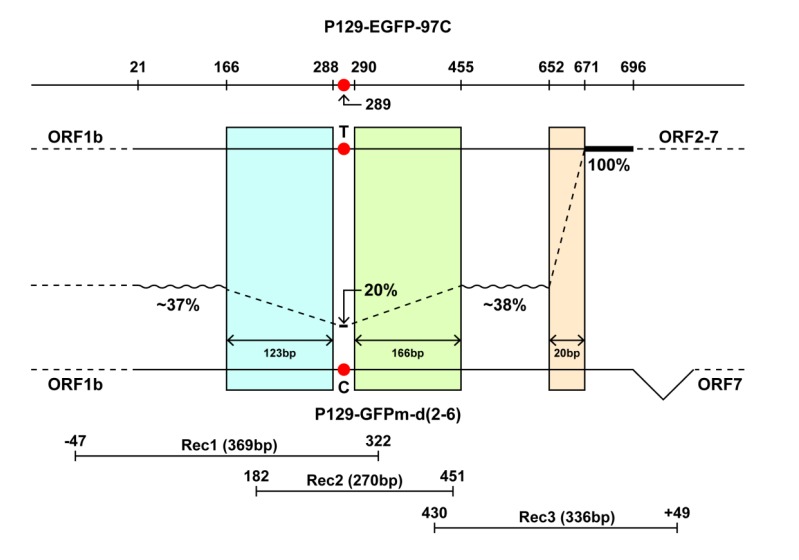
Evidence for the occurrence of cross-over events. The percentages of nucleotide identity to EGFP gene decreased from 37% (21bp-165bp variable region) to 20% (C_289_T substitution), then increased to 38% (456bp-651bp variable region) and to 100% (672bp-696bp variable region). The changes indicated that cross-over events occurred in three conserved regions: 166bp-288bp, 290bp-455bp, and 652bp-671bp.

The third conserved region (652-671nt) shows to be a cross-over hotspot and has higher cross-over rate than the above two conserved regions (Fig. **2**). The result is consistent with Sanger sequencing results that all six individual clones of the whole fluorescent gene from the same co-infection sample had cross-over at the 20bp-conserved region [20]. Although both RNA secondary structure and the length of sequence identity are crucial molecular determinants for recombination [5, 6, 28], RNA secondary structure seems to play a more important role in this case considering that the length of the cross-over hotspot (20bp) is shorter than the other two cross-over regions (123bp and 166bp). In addition, the 20bp-conserved region encoded “117-HMVLLE-222” is located at the junction region of β-sheet strand 11 linking to the loop. Glu222 at the junction point has alternative conformations [30]. Previous studies implied that recombination events occurred more frequency at the transcriptional pausing sites or polymerase-binding motifs [5, 17]. Therefore, the mechanisms responsible for the hotspot of the 20bp-conserved region are probably associated with RNA secondary structure. Notably, recombination events were not detected in the 20bp region when using infectious clones containing EGFP-97C and GFP, sharing 83% nucleotide identity [20]. Recombination events occurred between EGFP-97C and GFPm, which share 91% nucleotide identity, suggesting that the overall nucleotide identity may be also essential to produce recombinants. And another explanation is that the relative positions of the EGFP sequence inserted in GFPm gene may be important [6].

**Fig. (3):**
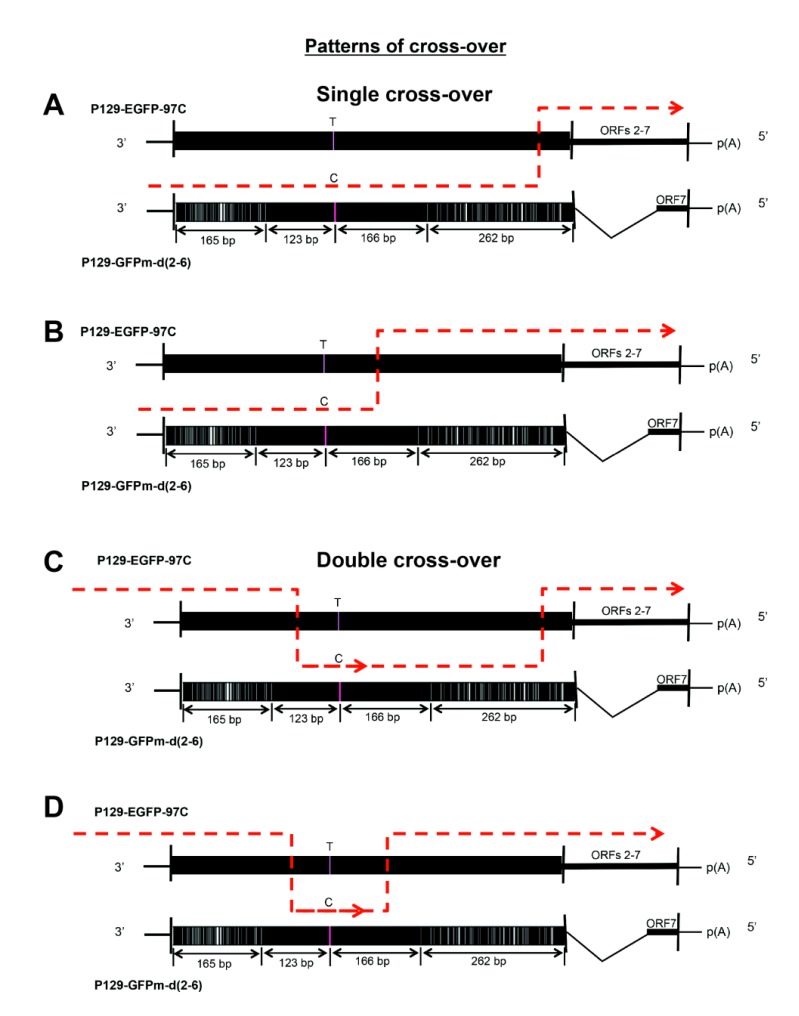
Four potential cross-over patterns used for producing recombinants. Two single cross-over Figs. (**3A** and **B**) and two double cross-over Figs. (**3C** and **D**) could be used to generate the viable fluorescent recombined viruses in this *in vitro* reverse genetics system.

**Fig. (4):**
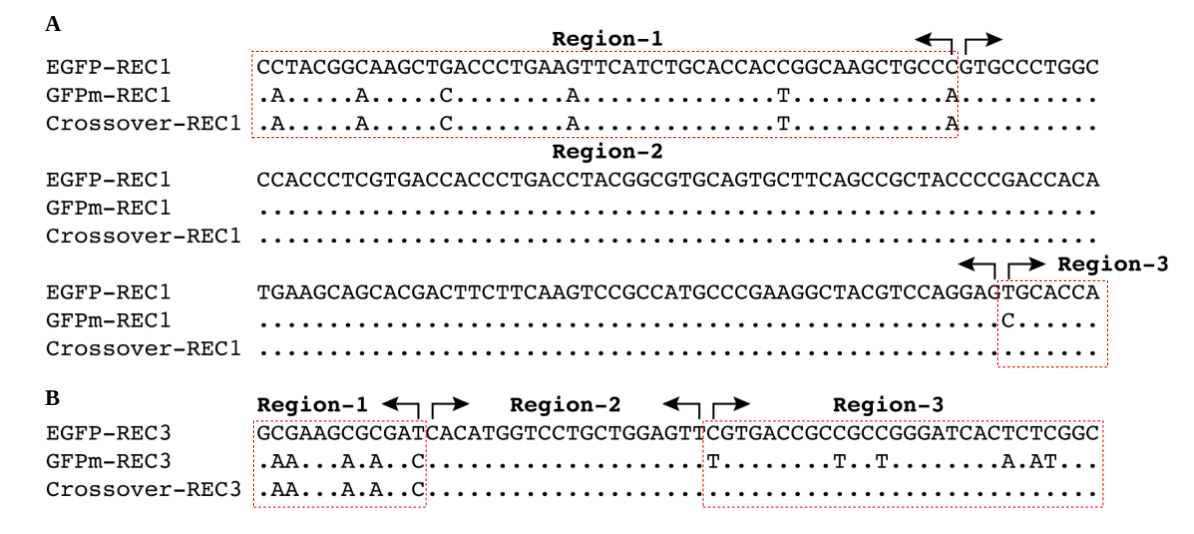
The alignment analysis identified two cross-over events. The cross-over events in the 123bp conserved region (4A) and 20bp conserved region (4B) were identified. The recombinants are identical to GFPm in region-1 but identical to EGFP in region-3, which were highlighted in red dotted line box. The conserved and cross-over regions are in region-2.

Four recombination patterns might be used to produce viable recombinants during co-infection (Fig. **3**). The exact rate of each pattern could not be determined due to the limitation of the read length (~400bp) of the 454 sequencing method used in this study, but two cross-over events were confirmed by sequence alignments (Fig. **4**). The results indicated that different cross-over patterns were utilized and distinct recombination events occurred simultaneously in the viral quasispecies.

## CONCLUSION

This study presented the detailed cross-over map of the chimeric genes in two PRRSV infectious clones by high throughput sequencing. Pattern analyses and sequence alignments indicated that different patterns may be utilized and distinct cross-over events may occur simultaneously. Our results also showed that the 20bp-conserved region is likely a cross-over hotspot, suggesting that RNA secondary structure may play a more important role for recombination in the case. This new *in vitro* reverse genetics system accompanied with deep sequencing creates a viable platform to study different types of recombination events and contributes to understanding of the requirements for recombination.

## Figures and Tables

**Table 1:** Primers used in this study.

PCR	Name	Sequence*(5'-3')
First round	Univ-A-Rec-F1	**TCTCGGTTCTGCATTCGA**CCCCGTCATTGAACCAACTTT
	Univ-B-Rec-R1	**ATTCGCTGGCACGCACTT**TGTAGTTGCCGTCGTCCTTGA
	Univ-A-Rec-F2	**TCTCGGTTCTGCATTCGA**TCGTGACCACCCTGACCTAC
	Univ-B-Rec-R2	**ATTCGCTGGCACGCACTT**CGTTGTGGCTGTTGTAGTTGTA
	Univ-A-Rec-F3	**TCTCGGTTCTGCATTCGA**TACAACTACAACAGCCACAACG
	Univ-B-Rec-R3	**ATTCGCTGGCACGCACTT**TGTTCCGCTGAAACTCTGGT
Second round	A-KMID1-Univ-A	**CCATCTCATCCCTGCGTGTCTCCGAC***TCAG*ACACGACGACT**TCTCGGTTCTGCATTCGA**
	B-K-Univ-B	**CCTATCCCCTGTGTGCCTTGGCAGTC***TCAG***ATTCGCTGGCACGCACTT**
* The universal tails are highlighted in bold, 454 adaptor sequences are bold and underlined, the key sequences are italic, the multiplex identifier (MID) is underlined, and targeted gene sequences are shown in regular.		

**Table 2:** Mutations and their percentages identified by comparing to GFPm gene.

Amplicon	Location*	Reference nucleotide (GFPm)	Variant nucleotide (EGFP)	Total Depth	Variant Frequency
>Rec1-EGFP	21	A	G	7182	37%
>Rec1-EGFP	30	T	C	7093	37%
>Rec1-EGFP	33	C	G	7086	37%
>Rec1-EGFP	39	C	G	7040	37%
>Rec1-EGFP	48	C	G	6797	35%
>Rec1-EGFP	51	G	C	6731	34%
>Rec1-EGFP	54	A	G	6716	34%
>Rec1-EGFP	60	T	C	6683	34%
>Rec1-EGFP	66	T	C	6315	31%
>Rec1-EGFP	117	A	C	6437	41%
>Rec1-EGFP	123	A	C	6425	41%
>Rec1-EGFP	129	C	G	6416	39%
>Rec1-EGFP	138	A	G	6403	39%
>Rec1-EGFP	153	T	C	6343	38%
>Rec1-EGFP	165	A	C	6300	36%
**>Rec2-EGFP**	**289**	**C**	**T**	**15531**	**20%**
*>Rec3-EGFP*	*456*	*C*	*T*	*15566*	*29%*
*>Rec3-EGFP*	*474*	*A*	*G*	*14345*	*37%*
*>Rec3-EGFP*	*480*	*T*	*C*	*14178*	*39%*
*>Rec3-EGFP*	*492*	*C*	*G*	*13736*	*43%*
*>Rec3-EGFP*	*505*	*A*	*C*	*13035*	*45%*
*>Rec3-EGFP*	*507*	*A*	*C*	*13023*	*45%*
*>Rec3-EGFP*	*514*	*A*	*G*	*12684*	*39%*
*>Rec3-EGFP*	*607*	*T*	*A*	*8032*	*37%*
*>Rec3-EGFP*	*608*	*C*	*G*	*8029*	*37%*
*>Rec3-EGFP*	*618*	*T*	*C*	*8407*	*36%*
*>Rec3-EGFP*	*633*	*T*	*C*	*8606*	*35%*
*>Rec3-EGFP*	*641*	*A*	*C*	*8598*	*36%*
*>Rec3-EGFP*	*642*	*A*	*G*	*8598*	*36%*
*>Rec3-EGFP*	*646*	*A*	*C*	*8711*	*35%*
*>Rec3-EGFP*	*648*	*A*	*C*	*8706*	*35%*
*>Rec3-EGFP*	*651*	*C*	*T*	*8698*	*35%*
>Rec3-EGFP	672	T	C	8616	100%
>Rec3-EGFP	681	T	C	8601	100%
>Rec3-EGFP	684	T	C	8591	100%
>Rec3-EGFP	693	A	T	8643	100%
>Rec3-EGFP	695	A	T	8648	100%
>Rec3-EGFP	696	T	C	8646	100%
* The locations of mutations are determined according to EGFP sequence. Mutations in the first variable region (21bp-165bp) are shown in regular. The C_289_T mutation is highlighted in bold and underlined. Mutations in the second variable region (456bp-651bp) are marked in italic. Mutations in the third variable region (672bp-696bp) are underlined.					
